# Doxycycline and Benznidazole Reduce the Profile of Th1, Th2, and Th17 Chemokines and Chemokine Receptors in Cardiac Tissue from Chronic* Trypanosoma cruzi*-Infected Dogs

**DOI:** 10.1155/2016/3694714

**Published:** 2016-09-01

**Authors:** Guilherme de Paula Costa, Laís Roquete Lopes, Maria Cláudia da Silva, Aline Luciano Horta, Washington Martins Pontes, Cristiane M. Milanezi, Paulo Marcos da Mata Guedes, Wanderson Geraldo de Lima, Richard Schulz, João Santana da Silva, Andre Talvani

**Affiliations:** ^1^Programa de Pós-Graduação em Ciências Biológicas/NUPEB, Universidade Federal de Ouro Preto, Ouro Preto, MG, Brazil; ^2^Faculdade de Medicina de Ribeirão Preto, USP, Ribeirão Preto, SP, Brazil; ^3^Universidade Federal do Rio Grande do Norte Federal, Natal, RN, Brazil; ^4^Departamento de Ciências Biológicas, Universidade Federal de Ouro Preto, Ouro Preto, MG, Brazil; ^5^Departments of Pediatrics & Pharmacology, Cardiovascular Research Centre, University of Alberta, Edmonton, AB, Canada; ^6^Programa de Pós-Graduação em Saúde e Nutrição, Universidade Federal de Ouro Preto, Ouro Preto, MG, Brazil

## Abstract

Chemokines (CKs) and chemokine receptors (CKR) promote leukocyte recruitment into cardiac tissue infected by the* Trypanosoma cruzi*. This study investigated the long-term treatment with subantimicrobial doses of doxycycline (Dox) in association, or not, with benznidazole (Bz) on the expression of CK and CKR in cardiac tissue. Thirty mongrel dogs were infected, or not, with the Berenice-78 strain of* T. cruzi *and grouped according their treatments: (i) two months after infection, Dox (50 mg/kg) 2x/day for 12 months; (ii) nine months after infection, Bz (3,5 mg/kg) 2x/day for 60 days; (iii) Dox + Bz; and (iv) vehicle. After 14 months of infection, hearts were excised and processed for qPCR analysis of Th1 (CCL2, CCL3, CCL4, CCL5, CXCL9, and CXCL11), Th2 (CCL1, CCL17, CCL24, and CCL26), Th17 (CCL20) CKs, Th1 (CCR5, CCR6, and CXCR3), and Th2/Th17 (CCR3, CCR4, and CCR8) CKR, as well as IL-17.* T. cruzi *infection increases CCL1, CCL2, CCL4, CCL5, CCL17, CXCL10, and CCR5 expression in the heart. Dox, Bz, or Dox + Bz treatments cause a reversal of CK and CKR and reduce the expression of CCL20, IL-17, CCR6, and CXCR3. Our data reveal an immune modulatory effect of Dox with Bz, during the chronic phase of infection suggesting a promising therapy for cardiac protection.

## 1. Introduction

Chemokines are low molecular weight proteins that promote leukocyte trafficking in homeostasis and inflammatory processes. Chronic inflammation is usually characterized by the persistence of leukocyte infiltration into the inflammatory site driven by chemokine production [1–3]. In particular,* Trypanosoma cruzi* infection is a well-known parasite disease where the presence of this protozoan triggers activation and continued leukocyte infiltration into the muscle tissues in order to eliminate it [4–6]. Since the stimulus persists over months, years, or decades, cell infiltration conducted by Th1 and Th2-like chemokines and their receptors may lead to loss of tissue's architecture and function, in some cases causing severe disability, especially in the heart [7, 8].

After the interaction between* T. cruzi* and mammalian host cells, the activation of innate resident macrophages/neutrophils combined with the mobilization of natural killer cells, TCD4+, TCD8+, and gamma-delta T cells releases vasoactive substances and inflammatory mediators triggered by the invasion of blood trypomastigote forms of this parasite [9, 10]. Latter, IL-12 induces a Th1-type differentiation, mediated by IFN-*γ*, TNF, and IL-17, culminating in NO biosynthesis by macrophages and in the generation of a panel of inflammatory chemokines that orchestrates and accelerates acute immunopathogenesis [11–13].

On the other hand, chronic cardiac inflammation induced by* T. cruzi* is a harmful and silent event that occurs due to the failure in resolving the acute inflammation and/or due to the persistence of the parasitic stimulus. The involvement and importance of CK and CKR to the generation of the chronic cardiomyopathy induced by* T. cruzi* were previously highlighted by our group, in humans and in experimental animals (rodent and dog) [12, 14–17]. Even at this chronic stage, IFN-*γ* and TNF persist as essential cytokines, maintaining the migration of T cells to consolidate the cardiomyopathy by increasing the expression of CCL5 (RANTES), CCL2 (MCP-1), CXCL10 (IP-10), and CXCL9 (MIG) as well as enhancing intercellular adhesion and vascular cell adhesion molecules [7, 12]. Comprehending the role of CK and CKR on immune cells, from acute to chronic cardiac disease, may open new therapies to reduce the damage caused by chronic* T. cruzi*-induced cardiomyopathy.

In this present study, Dox, a member of the tetracycline family, was investigated based on its capacity to selectively inhibit matrix metalloproteinases and modulate immune response, already at a subantimicrobial dose [18–21]. Dox was tested alone and in combination with the antiparasitic drug, benznidazole (BZ) which also possesses a potential immune modulatory role [22–24]. Assuming the importance of leukocyte recruitment to* T. cruzi*-induced myocarditis, both pharmacological interventions were investigated for their potential interference on the expression of cardiac CK and CKR receptors in dogs infected with the Berenice-78 strain of* T. cruzi* [25], an experimental model with close similarity to human Chagas myocarditis [26, 27].

## 2. Materials and Methods

### 2.1. Animals, Infection, and Treatments

Thirty mongrel dogs of either sex (4 months old), obtained from the Animal Facility at Universidade Federal de Ouro Preto (UFOP, MG, Brazil), were infected or not with 2 × 10^3^ bloodstream forms of Berenice-78 (Be-78) strain of* Trypanosoma cruzi*. The animals were fed with a commercial dog food and water* ad libitum*. Prior to the study they were vaccinated and dewormed against several parasitic diseases.

Male and female animals were grouped (*n* = 5) according to their treatment: (i) doxycycline (Dox) (50 mg/kg) twice a day for 12 months starting at 2 months after* T. cruzi* infection, (ii) benznidazole (Bz) (3,5 mg/kg) twice a day for 60 days starting at the 9th month of infection, (iii) Dox + Bz, and (iv) vehicle (0.5% carboxymethylcellulose, in water). Dox has plasma half-life of 10–12 h, and antimicrobial dosing requires 100–200 mg twice/daily [18]. In parallel, an uninfected group was evaluated during the period of experimentation. Animals were gently immobilized according to the guidelines of the Brazilian College of Animal Experimentation (COBEA), and the administration of medicines and vehicle was performed by a direct injection of 3 mL of the solution in the oropharynges of the animals using a plastic syringe; the solution was diluted in a soup to facilitate drug administration.

The animals were euthanized at the 14th month after infection and fragments of the left ventricle were stored in TRIzol® at −80°C. All animal experiments and procedures were performed in accordance with the COBEA and approved by the Ethical Committee for Experiments with Laboratory Animals at UFOP (CEUA-UFOP/Protocol number 2013/60).

### 2.2. RNA Extraction and cDNA Synthesis

Total RNA from cardiac tissues (30–35 mg of left ventricle) were isolated using 0.5 mL of TRIzol reagent (Invitrogen) or the SV Total RNA Isolation System (Promega, Madson, WI) according to manufacturer's instructions. The RNA yield and the ratio of absorbance at 260–280 nm (*A*_260_/*A*_280_ ratio) were measured using a NanoVue Plus Spectrophotometer (GE Healthcare, UK). Samples containing <10 ng/*µ*L of RNA were excluded from the study. The cDNA was synthesized using 1 *µ*g of total RNA through a reverse transcription reaction (Reverse Transcriptase, Promega). The cDNA *β*-actin copy number/RNA *β*-actin copy number ratio was calculated as a measure of the efficiency of the cDNA synthesis; this ratio was used to normalize the reference gene copy numbers as assessed by quantitative real-time PCR.

### 2.3. Development of Real-Time PCR Assays for Canine CK and CKR

For the CK and CKR genes, a standard curve from serial dilutions of a known concentration of purified DNA was achieved. This quantified DNA consists of the target PCR product prepared by conventional PCR, from cDNA positive for the corresponding target mRNA. Threefold measurements, for each standard dilution point over the whole standard curve range, produced to generate a reliable standard curve. Then, real-time PCR quantitative mRNA analyses were performed using an ABI Prism 7000 SDS unit (Applied Biosystems) through the Platinum® SYBR® Green qPCR SuperMix UDG with ROX reagent (Invitrogen) for quantification of amplicons. The standard PCR conditions were as follows: 50°C (2 min), 95°C (10 min); 40 cycles of 94°C (30 s), 58°C (30 s), and 72°C (1 min), followed by the standard denaturation curve, as performed previously by our group [16]. The sequences of the primers were designed using the Primer Express software (Applied Biosystems) assuming the nucleotide sequences available in the GenBank database (Table 1). In each reaction the Platinum® SYBR® Green qPCR SuperMix UDG with ROX reagent (Invitrogen), 1 *µ*g/*µ*L of each specific primer, and cDNA diluted 20 times were used. In this study, all data were normalized to beta-actin mRNA. Relative increases in CK and CKR were plotted in comparison to the noninfected control group using 2ΔΔCT method.

### 2.4. Histopathology

Animals were euthanized at the 14th month after infection and heart tissue fragments with an area of 0.2 cm^2^ from the middle of the left ventricle wall were taken for histopathology analysis. Tissue fragments were fixed in 10% buffered formalin solution, dehydrated, cleared, and embedded in paraffin. Blocks were cut into 4 mm thick sections and stained by hematoxylin and eosin (H&E) for inflammation assessment. Twenty fields from each hematoxylin and eosin stained sections were randomly chosen at a 40x magnification, giving an area of 1,5 × 10^6^ *μ*m^2^ of analyzed myocardium. Images were obtained through a Leica DM 5000 microchamber (Leica Application Suite, version 2.4.0R1) and processed by Leica Qwin (V3) image analyzer software. The inflammatory process was estimated using a correlation index of the number of cell nuclei observed in the myocardium from uninfected animals. This index corresponds to the number of nuclei of inflammatory leukocytes plus the background of cardiac cells nuclei observed in the uninfected dog samples.

### 2.5. Statistical Analysis

Data are expressed as the mean ± standard error of the mean (SEM) and were analyzed using the* Kolmogorov-Smirnov* normality test and One-Way analysis of variance. All analyses were performed using PRISM 5.01 software (GraphPad, San Diego, CA, USA) and the level of significance was accepted at *p* < 0.05.

## 3. Results

### 3.1. Chemokine mRNA Expression in Cardiac Tissues from* T. cruzi*-Infected Animals

**Figure 1:**
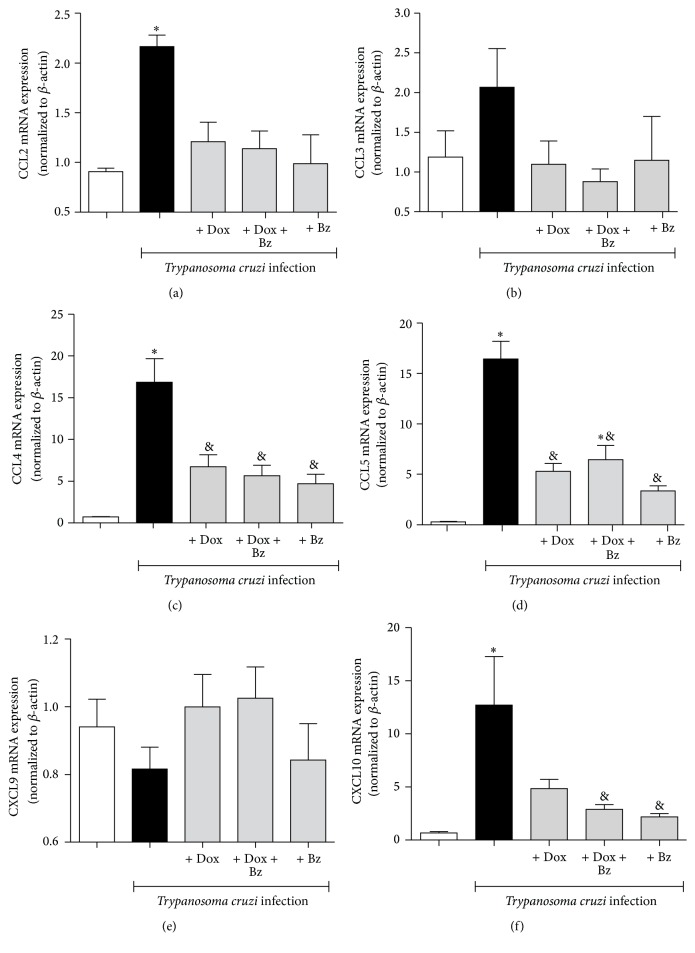
Expression of Th1-like chemokines in the cardiac tissue from dogs infected with* Trypanosoma cruzi*. Mongrel dogs were infected (*black bars*), or not (*white bars*), with Berenice-78 strain of* T. cruzi* and treated daily for 12 months with doxycycline (Dox), for 60 days with benznidazole (Bz), or in association with Dox + Bz (*grey bars*) and Th1-like chemokines CCL2, CCL3, CCL4, CCL5, CXCL9, and CXCL10 mRNA expression evaluated in left ventricle tissues from these animals. Data are representative of groups of 5 animals in which expression of chemokines was normalized to the constitutive beta-actin. ^*∗*^ *p* < 0.05 in relation to the noninfected group; ^&^ *p* < 0.05 in relation to the infected group without treatment.

The mRNA expression of the Th1 profile chemokines, able to recruit T cells, monocytes, and memory T cells, was evaluated in left ventricle from chronically* T. cruzi*-infected dogs. The parasite was able to induce a high expression of CCL2 (Figure 1(a)), CCL4 (Figure 1(c)), CCL5 (Figure 1(d)), and CXCL10 (Figure 1(f)) when compared to uninfected control animals. At this stage of infection, only CCL3 (Figure 1(b)) and CXCL9 (Figure 1(e)) showed no changes in mRNA expression. In the presence of a long-term treatment with Dox alone or in association with Bz, the mRNA levels of CCL4 and CCL5 were reduced. This profile was also observed in monotherapy with Bz for 60 days, starting at the chronic phase.

**Figure 2:**
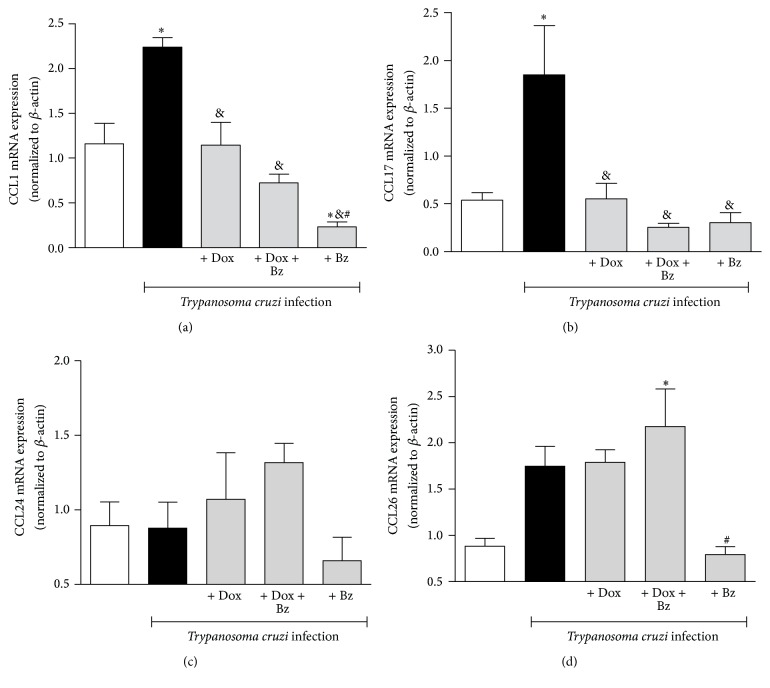
Expression of Th2-like chemokines in the cardiac tissue* Trypanosoma cruzi*-infected dogs. Th2-like chemokines CCL1, CCL17, CCL24, and CCL26 mRNA expression evaluated in left ventricle tissues. Data are representative of the groups (*n* = 5 animals) in which the expression of chemokines was normalized to the constitutive beta-actin. *∗* means difference (*p* < 0.05) in relation to the noninfected group; & means difference (*p* < 0.05) in relation to the infected group without treatment; # means difference (*p* < 0.05) in relation to the Dox and Dox + Bz.

**Figure 3:**
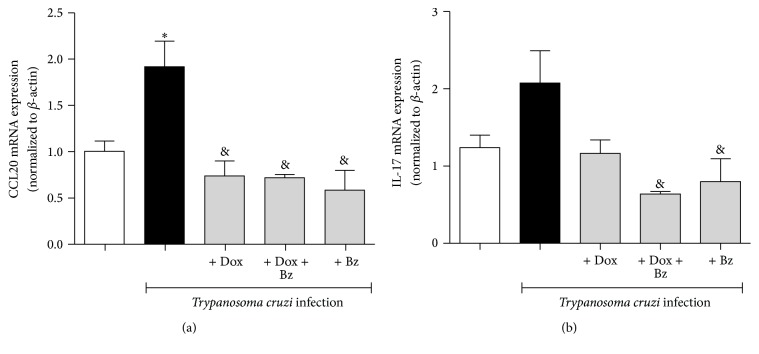
Expression of Th17-like chemokine CCL20 and IL-17 in the cardiac tissue from dogs infected with* Trypanosoma cruzi*. CCL20 and IL-17 mRNA expression evaluated in left ventricle tissues. Data are representative of groups (*n* = 5 animals) in which expression of chemokines was normalized to the constitutive beta-actin. & means difference (*p* < 0.05) in relation to the infected group without treatment; *∗* means difference (*p* < 0.05) in relation to the noninfected group.

The profile of Th2-like chemokines was also evaluated. An increase in the mRNA expression of CCL1 (Figure 2(a)) and CCL17 (Figure 2(b)) in cardiac tissue from infected dogs was seen. The expression of Th2-like chemokines, involved with the recruitment of monocytes and T cells, reduced after Dox, Dox + Bz, or Bz therapies. However, the Th2-like chemokines CCL24 (Figure 2(c)) and CCL26 (Figure 2(d)), both involved in eosinophil recruitment, were not enhanced in the presence of the parasite. The associative Dox + Bz therapy was able to increase the expression of the CCL26 (Eotaxin-3) whereas monotherapy with Bz showed an opposite effect. The Th17-like chemokine CCL20 (MIP-3b) which acts on regulatory and memory T cells as well as dendritic and B cells also showed high mRNA expression in the left ventricle tissue from* T. cruzi*-infected animals (Figure 3(a)). Therapies with Dox, Dox + Bz, or Bz were all able to reduce the expression of CCL20. However, IL-17, which has an important role in experimental acute infection with* T. cruzi*, was not significantly enhanced in the chronic phase of infection with this strain of* T. cruzi*. (Figure 3(b)).

### 3.2. The CKR Receptors mRNA Expression in Cardiac Tissues from* T. cruzi* Infected Animals

**Figure 4:**
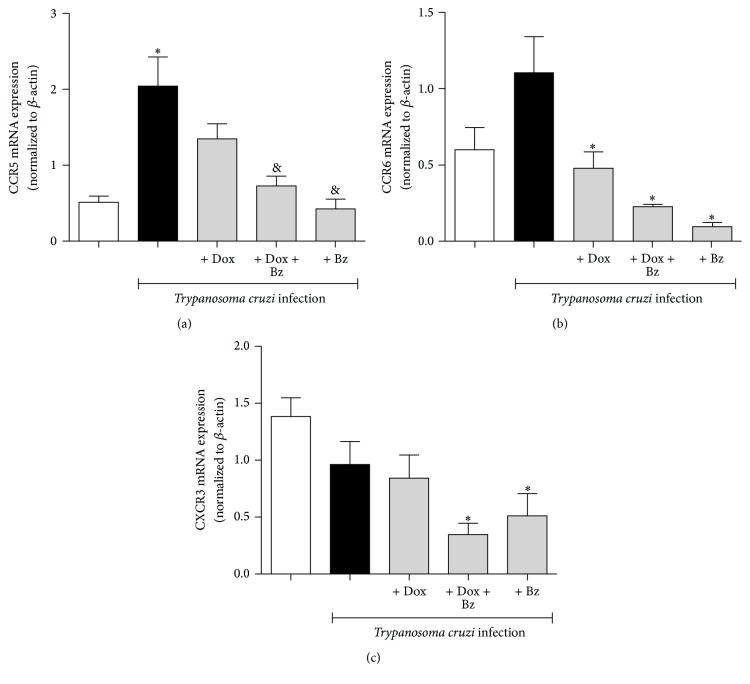
Expression of Th1-like chemokine receptors in the cardiac tissue* Trypanosoma cruzi*-infected dogs. Th1-like chemokine receptors (CCR5, CCR6, and CXCR3) mRNA expression evaluated in the left ventricle tissues. Data are representative of the groups (*n* = 5 animals) in which the expression of chemokine receptors was normalized to the constitutive beta-actin. *∗* means difference (*p* < 0.05) in relation to the noninfected group; & means difference (*p* < 0.05) in relation to the infected group without treatment.

The mRNA expression of CKR with Th1 profile, such as CCR5 (Figure 4(a)), whose ligands include CCL3, CCL4, CCL5, CCL11, CCL14, and CCL16, was significantly enhanced in the left ventricle from infected dogs. Bz reduced this expression, whether alone or in combination with Dox, than monotherapy. The other chemokine receptors with Th1 profile evaluated in this study, CCR6 (Figure 4(b)) and CXCR3 (Figure 4(c)), showed no significant changes following* T. cruzi* infection. However, for both receptors, the combination or the monotherapies were able to decrease their mRNA expression, less than in the uninfected dogs, except to the CXCR3 in dogs under Dox treatment (Figure 4(c)).

**Figure 5:**
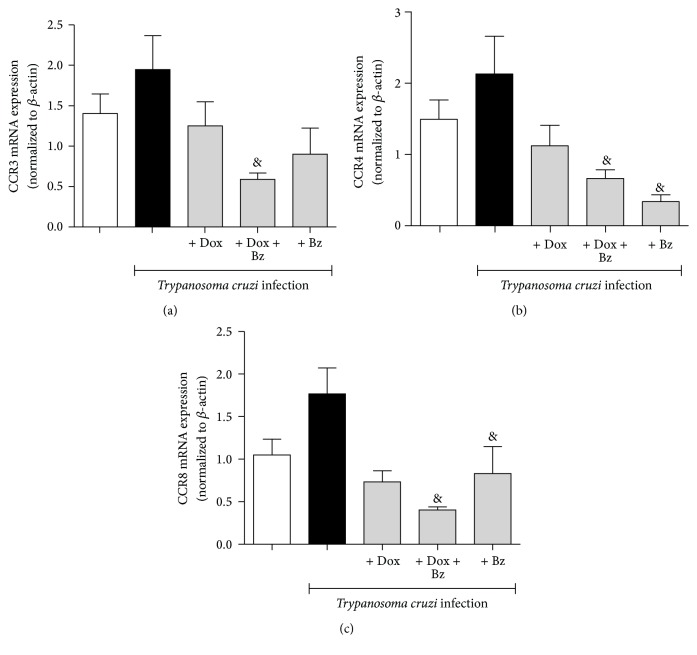
Expression of Th2/Th17-like chemokine receptors in the cardiac tissue from dogs infected with the* Trypanosoma cruzi*. Th2/TH17-like chemokine receptors (CCR3, CCR4, and CCR8) mRNA expression evaluated in the left ventricle tissues. Data are representative of the groups (*n* = 5 animals) in which the expression of chemokine receptors was normalized to the constitutive beta-actin. & means difference (*p* < 0.05) in relation to the infected group without treatment.

In partial accordance with the expression in the pattern of some Th2/Th17 CK, the expressions of Th2/Th17 profile of CKR CCR3 (Figure 5(a)), CCR4 (Figure 5(b)), and CCR8 (Figure 5(c)) were also unchanged in dogs infected. Even so, the combination therapy Dox + Bz reduced the mRNA expression of all CKR when compared with the level seen in infected cardiac tissues.

### 3.3. Leukocyte Infiltration in Left Ventricular Tissue

**Figure 6:**
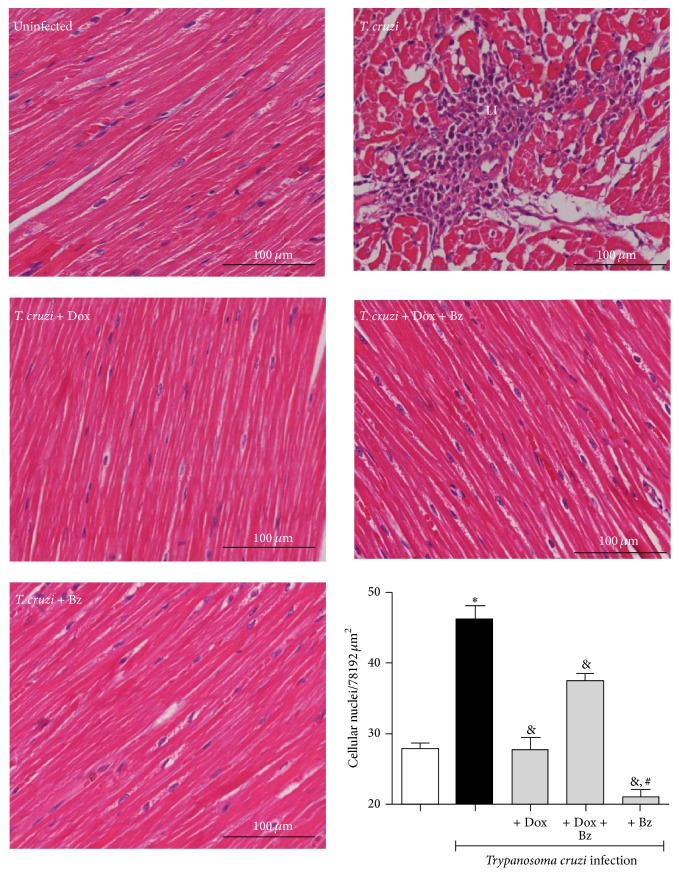
Benznidazole and doxycycline reduce inflammation in the* T. cruzi*-infected left ventricle. Mongrel dogs infected with* T. cruzi* and treated with doxycycline (Dox), with benznidazole (Bz), or with the association of Dox + Bz had their heart extracted in the chronic phase to evaluate the inflammatory infiltration (H&E) in the left ventricle segment. The noninfected group was evaluated in parallel and sections were quantified in sections with 40x of magnification. Data are representative of the groups (*n* = 5 animals) and ^*∗*^ *p* < 0.05 in relation to the noninfected group; ^&^ *p* < 0.05 in relation to the infected group without treatment; # means difference (*p* < 0.05) in relation to the Dox and Dox + Bz. LI: leukocytes infiltration.

CK and CKR were evaluated, in parallel, with the evidence of leukocyte infiltration in the left ventricle tissue, by estimating the total cell nuclei (cardiac cells and leukocytes infiltrated into the cardiac tissue). Chronic* T. cruzi*-infected dogs presented scattered infiltrating cells whereas there was an absence of detectable cellular infiltration in uninfected animals and in infected dogs treated with Dox for 12 months and with Bz for 60 days during the chronic infection phase (Figure 6), in monotherapy or in combination.

## 4. Discussion

In this present study, an associative and a long-term therapy with Bz and Dox was proposed to mongrel dogs infected with the Berenice-78 strain of* T. cruzi*. We observed a significant reduction of mRNA expression to the Th1 and Th2/Th17-like CK/CKR, as well as to IL-17 in the left ventricle tissue in association with Bz and Dox therapies. This fine tuning between pharmacological intervention and CK/CKR expression may result in a gradual protection with low inflammatory infiltration, low cellular destruction, and cardiac architecture preservation.

The concepts of* T. cruzi*-induced inflammation as the trigger of cardiac remodeling are essential since parasites can persist in the host tissues. Bz is the main drug currently used to eliminate this parasitic infection; however, its effectiveness is observed only when administered during the initial stage of infection [28, 29]. New pharmacological strategies have been proposed in experimental models aiming at not only eliminating the parasite but also modulating the inflammatory mediators through the reduction of the release and expression of cytokines and CKs, systemically and in the infected heart tissue.

The relevance of the chemokine network to the generation and progression of the cardiomyopathy induced by* T. cruzi* has gained strong evidence in different experimental models as well as in studies involving individuals with chronic Chagas cardiomyopathy [8, 30–32]. After the* T. cruzi* invasion, activation of the resident immune and endothelial cells precedes the enhanced expression of adhesion molecules, intensifying the recruitment of monocytes, neutrophils, and T cells towards infected muscle, in particular into the cardiac tissue [33, 34]. CKs and their receptors have awaken the immune system towards this infected environment, worsening the pathological cardiac condition and perpetuating it, since the antigenic stimuli are usually not completely eliminated.

Dogs infected with Berenice-78* T. cruzi* strain were previously described by de Lana et al. [26], highlighting this “dog model” as the ideal to study experimental* T. cruzi*-induced cardiomyopathy due to the similarity between clinical and immune parameters with those observed in humans. In particular, the Berenice-78 strain of* T. cruzi* induces a prominent focal and diffuse fibrosis in the myocardium. In Berenice-78 infected dogs, the background of the inflammatory status during the chronic phase does not appear to be dependent on IL-17 or CCL20/CCR6. This latter ligand-receptor pair exerts chemoattraction to B cells, immature dendritic cells, effector, and memory T cells and also plays a role at the skin, mucosal, and intestinal immune system [35]. IL-17 is an essential mediator acting to resolve* T. cruzi* infection in mice by increasing STAT-3 and RORgt mRNA, which reflects on the expression of Th1-type chemokines involved with T cell recruitment (CCL2, CCL3, CCL4, CCL11, and CXCL9) [36]. IL-17 produced by TCD4+, TCD8+, and NK cells are, in part, induced by trypomastigote forms of the parasite in the murine model which suggests its participation centered on the acute phase of infection. Concerning the CKs with Th2-profile, there was an increased expression of CCL1 and CCL17 in infected left ventricles. This CCL1-CCR8 axis is linked to the adaptive immune response, possibly influenced by GPI-mucin antigenic stimuli from* T. cruzi* during the recent chronic phase of infection [37]. Similarly, CCL17, but not its shared receptor CCR4, was also enhanced in these infected dog hearts. This Th2 chemokine, which participates in the development of local fibrosis and in atherosclerotic plaque inflammation, may act to locally constrain the regulatory T cells (Treg) maintenance and thereby intensify vascular inflammation [38].

The Th1-type CKs are the most important mediators expressed in the myocardium of dogs infected with the Berenice-78 strain of* T. cruzi*. Similar findings were demonstrated in human hearts, where the mRNA expressions of the CKs CCL5, CXCL9, CXCL10, CCL17, CCL19, and their receptors were upregulated in chronic Chagas cardiomyopathy [31]. The enhancement of CCR5 expression with its ligands CCL3, CCL4, and CCL5 in the parasite infected tissue is consistent with its important role in promoting leukocyte (CD8+ T cells and monocytes) infiltration within target organs as well as limiting parasite replication during early* T. cruzi* infection [39]. Leukocytes from individuals with chronic Chagas cardiomyopathy showed high expression of CCR5 and low expression of CXCR4 and this pattern clearly correlates with heart function [15]. In addition, polymorphism in the CCR5-2733 and CCR5-2554 T alleles was associated with the reduced risk of susceptibility and severity to the development of Chagas cardiomyopathy, respectively [40]. Expressions of CKs and their receptors have been shown to be important prognostic markers, since CCL5 and CCL9 chemokine genes were upregulated in heart tissue from individuals with Chagas cardiomyopathy [41]. Of note, similar studies in beagle dogs reinforced that mRNAs of CKs with a Th1-like profile (CCL4, CCL5, CCL26, and CXCR3) are overexpressed in animals presenting chronic cardiomyopathy [16] as well in this present study using chronic* T. cruzi*-infected mongrel dogs where higher expressions of CCL2, CCL4, CCL5, CXCL10, and CCR5 were also observed in the left ventricle associated with low ejection fraction (data not shown). These data are consistent with locally released Th1-CKs assisting in the accumulation of Th1 T cells into the* T. cruzi*-infected cardiac tissue previously described in humans and in animal models.

However, the highlighted and most promising discovery from this study is the use of Dox as a modulator of the CK network in infected cardiac tissue. Doxycycline is noted for its significant effect on its dose-dependent modulation of inflammatory mediators (TNF, IL-1beta, IL-6, IL-8, CCL2, CCL3, and CCL4) in different types of cell lines [42, 43]. Previous studies supported its property to inhibit T cell proliferation and the cytokine/CK release in peripheral blood mononuclear cells stimulated by staphylococcal exotoxins stimuli [44] and also to prevent lipopolysaccharide-induced endothelial barrier dysfunction by inhibiting the activation of the p38/MAPK pathway in endothelial cells from human umbilical vein [45]. In some human disorders where inflammatory status and protease activities go hand-in-hand as in osteoarthritis, angiogenesis, ulcers, and others, the beneficial effects of doxycycline are not due to its antimicrobial properties, but rather its ability to inhibit matrix metalloproteinase activities. In note, besides the already described inhibitory effects of the doxycycline on matrix metalloproteinase in hearts from* T. cruzi*-infected mice [46], here we hypothesized that Dox was acting, in the infected mongrel dogs, through the cytokines/CK production reducing cardiac inflammation and the clinical repercussions at the end-stage of this infection.

## 5. Conclusions

In summary, our data demonstrates the importance of therapeutic, long-term intervention with submicrobial dosing of Dox in association with chronic treatment with Bz in order to decrease or retard the development of* T. cruzi*-induced cardiac injury through the modulation of the expression of the Th1- and Th2/Th17-like CK/CKR axis in the cardiac left ventricle. In addition, this study reinforces the need for the discovery of new therapies with less toxicity, not only focusing on parasite elimination but also modulating the immune response against cardiac chronic infection. This harmonic equilibrium may be essential to prevent adverse cardiac remodeling as well as to increase the lifespan of individuals infected with the* T. cruzi*.

## Figures and Tables

**Table 1:** Primer sequences according to the GenBank database.

	Sequences (forward and reverse)
CCL2	TAAAAGAGTCACCAGCAGCAA
	TTTAGGACGGTCTTGAAGATCA
CCL5	CAAGCAGATTCCACGCAAGTT
	TAATACCGGGCTTGGAGCAT
CCL4	TGACCGTCCTTTCTCTCCTT
	GATCTGAACCCATTGGTGCT
CCL5	AAGGGCTGACTGATAAATGTGA
	AGCGAGAATTTTAATGGAAAGC
CCL17	CCATCGTGTTTGTAACTGTCCA
	AATATCTGACCGCCTTCTTCAC
CCL20	TATGGTTCCTCCCGGATCTATC
	TCATTGGCCAGCTGTTGTTGTGT
CCL24	CCTGCTGCATGTTCTTCATTTC
	TTCTGGTTCTTCTTGGTGGTGA
CCL26	TCTTCATCCTGAGTGTCCATCG
	AGCAGAACTTGGCCACATCA
CXCL9	CAGATGGTCCTTAAGCCACTTT
	CCTTTCCCTGTGAACCTCAA
CXCL10	CACATGTTGAGATCATTGCCAC
	TTCAGACATCTTTTCTCCCCA
CCR3	CTAGCAGCCTCCCCTGAATTTA
	TGTTCAGACTCCTTTTGGGACT
CCR4	TTTGGACTAGGTCTCTGCAAGA
	AAAAGCCCACCAGGTACATC
CCR5	TGTGTCTGCTTCAAAAGCCC
	TCACTTGTCACCACCCCAAA
CCR6	AGCTGTTTGTGCCAATTGCTTA
	AAAATATTGCCCAGGAGGCC
CCR8	TGATATCATCTCAAGCCCCTG
	AGCAACTTGCTGTCTCTTTGGA
CXCR3	TTCTTTGCCATCCCAGATTTC
	ATGCATGGCATTTAGGCG
IL-17	ACTCCAGAAGGCCCTCAGA
	GATTCCAAGGTGAGGTAGATCG
*β*-actin	CCACTTTCCTGTCTTACCCAA
	AATTAACCACCCACGGTGTT
